# Induction of Syndecan-4 by Organic–Inorganic Hybrid Molecules with a 1,10-Phenanthroline Structure in Cultured Vascular Endothelial Cells

**DOI:** 10.3390/ijms18020352

**Published:** 2017-02-08

**Authors:** Takato Hara, Takayuki Kojima, Hiroka Matsuzaki, Takehiro Nakamura, Eiko Yoshida, Yasuyuki Fujiwara, Chika Yamamoto, Shinichi Saito, Toshiyuki Kaji

**Affiliations:** 1 Department of Environmental Health, Faculty of Pharmaceutical Sciences, Tokyo University of Science, Noda 278-8510, Japan; 3b13669@ed.tus.ac.jp (T.H.); 3b14641@ed.tus.ac.jp (T.K.); 3b11082@ed.tus.ac.jp (H.M.); 3a15702@ed.tus.ac.jp (T.N.); eyoshida@rs.tus.ac.jp (E.Y.); 2 Department of Environmental Health, School of Pharmacy, Tokyo University of Pharmacy and Life Sciences, Hachioji 192-0392, Japan; yasuyuki@toyaku.ac.jp; 3 Department of Environmental Health, Faculty of Pharmaceutical Sciences, Toho University, Funabashi 274-8510, Japan; yamamoto@phar.toho-u.ac.jp; 4 Department of Chemistry, Faculty of Science, Tokyo University of Science, Shinjuku 162-8601, Japan; ssaito@rs.kagu.tus.ac.jp

**Keywords:** endothelial cells, proteoglycan, 1,10-phenanthroline, syndecan-4, bioorganometallics

## Abstract

Organic–inorganic hybrid molecules constitute analytical tools used in biological systems. Vascular endothelial cells synthesize and secrete proteoglycans, which are macromolecules consisting of a core protein and glycosaminoglycan side chains. Although the expression of endothelial proteoglycans is regulated by several cytokines/growth factors, there may be alternative pathways for proteoglycan synthesis aside from downstream pathways activated by these cytokines/growth factors. Here, we investigated organic–inorganic hybrid molecules to determine a variant capable of analyzing the expression of syndecan-4, a transmembrane heparan-sulfate proteoglycan, and identified 1,10-phenanthroline (*o*-Phen) with or without zinc (Zn-Phen) or rhodium (Rh-Phen). Bovine aortic endothelial cells in culture were treated with these compounds, and the expression of *syndecan-4* mRNA and core proteins was determined by real-time reverse transcription polymerase chain reaction and Western blot analysis, respectively. Our findings indicated that *o*-Phen and Zn-Phen specifically and strongly induced syndecan-4 expression in cultured vascular endothelial cells through activation of the hypoxia-inducible factor-1α/β pathway via inhibition of prolyl hydroxylase-domain-containing protein 2. These results demonstrated an alternative pathway involved in mediating induction of endothelial syndecan-4 expression and revealed organic–inorganic hybrid molecules as effective tools for analyzing biological systems.

## 1. Introduction

Vascular endothelial cells are located in a monolayer on the luminal surface and play a crucial role in regulating the blood coagulation-fibrinolytic system by synthesizing and secreting several physiological factors, including von Willebrand factor and tissue factors that promote blood coagulation [1,2], and prostacyclin, which suppresses platelet aggregation [3]. Additionally, tissue-plasminogen activator converts plasminogen to plasmin [4] capable of degrading fibrin, and proteoglycans that consist of core proteins and one or more glycosaminoglycan side chain(s) exist on the vascular endothelial cell surface and extracellular matrix [5]. Vascular endothelial cells synthesize and secrete two types of proteoglycans: heparan-sulfate proteoglycans and dermatan-sulfate proteoglycans. Perlecan is a large heparan-sulfate proteoglycan and a component of basement membranes [6], and the syndecan family of proteins includes transmembrane heparan-sulfate proteoglycans [7], whereas the glypican family of proteins includes glycosylphosphatidylinositol-anchored heparan-sulfate proteoglycans [6,7,8]. Vascular endothelial cells also synthesize and secrete small leucine-rich proteoglycans, including biglycan and decorin [9,10]. Furthermore, heparan- and dermatan-sulfate chains activate antithrombin III and heparin cofactor II, respectively, and contribute to the anticoagulant properties of vascular endothelium [8,11].

Clarification of the mechanisms involved in regulating proteoglycan expression is important for understanding the regulation of vascular endothelial cell functions. Various cytokines and growth factors regulate the expression of proteoglycans in vascular endothelial cells. Vascular endothelial growth factor-165 and fibroblast growth factor-2 (FGF-2) induce the expression of perlecan and biglycan, respectively [12,13]. When cell density is low, connective-tissue growth factor upregulates decorin expression, but downregulates biglycan expression [14]. Additionally, transforming growth factor-β_1_ enhances perlecan and biglycan expression in a cell-density dependent manner [15]. Recently, we reported that transforming growth factor-β_1_ initially upregulates and then downregulates the expression of syndecan-4 in vascular endothelial cells [16]. However, because it is likely that regulation of endothelial proteoglycan synthesis by growth factors/cytokines is mediated by downstream signaling pathway(s) associated with corresponding receptors, it may be difficult to discover alternative pathways involved in regulating endothelial proteoglycan synthesis by using growth factors/cytokines as analytical tools.

Organic–inorganic hybrid molecules are compounds consisting of ligand(s) and metal(s) and have been utilized as synthetic reagents for chemical reactions, thereby contributing to the development of organic synthetic chemistry. However, organic–inorganic hybrid molecules have not been widely used in biological studies. Recently, we reported that several organic–inorganic hybrid molecules show distinctive biological activities in cellular responses, with these activities—including cytotoxicity—being unrelated to either the organic structure or the inorganic metals forming the hybrid molecules [17,18,19]. This suggested that organic–inorganic hybrid molecules might be a good tool to analyze biological systems in certain cases. We subsequently confirmed this by successfully analyzing intracellular signaling pathways that mediate endothelial metallothionein induction using copper/zinc complexes [20,21].

As a preliminary study, we searched zinc complexes that modulate proteoglycan synthesis in vascular endothelial cells from a library of zinc complexes and discovered dichloro(1,10-phenanthroline)zinc (Zn-Phen) as an inducer of endothelial syndecan-4 synthesis. Based on this finding, we also prepared 1,10-phenanthroline (*o*-Phen), the ligand of Zn-Phen, and potassium tetrachloro(1,10-phenanthroline)rhodate(III) (Rh-Phen), which maximizes the strong binding capability between rhodium and *o*-Phen. In this study, we analyzed intracellular signaling pathways that mediate syndecan-4 expression in vascular endothelial cells using *o*-Phen, Zn-Phen, and Rh-Phen. Our results indicated that *o*-Phen, Zn-Phen, and Rh-Phen upregulated syndecan-4 expression in vascular endothelial cells, and that the strength of the upregulation was on the order of *o*-Phen > Zn-Phen > Rh-Phen. Furthermore, we used these compounds to investigate the intracellular signaling pathways that mediate the upregulation of endothelial syndecan-4 expression.

## 2. Results

### 2.1. o-Phen, Zn-Phen, and Rh-Phen Induce Syndecan-4 Expression in Vascular Endothelial Cells

**Figure 1:**
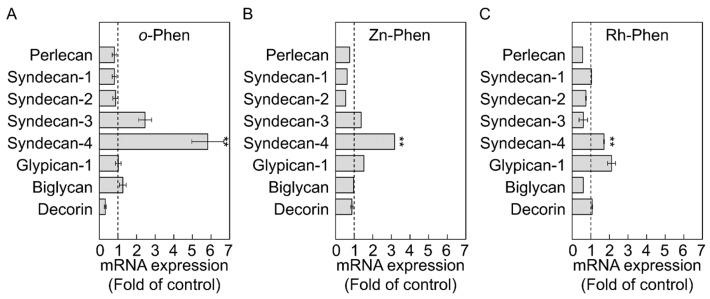
Effects of *o*-Phen, Zn-Phen, and Rh-Phen on proteoglycan expression in vascular endothelial cells. Bovine aortic endothelial cells were treated with *o*-Phen (**A**); Zn-Phen (**B**); or Rh-Phen (**C**) at 5 µM each at 37 °C for 24 h and proteoglycan mRNA levels were analyzed by real-time RT-PCR. Values are means ± S.E.M. of four samples. ** *p* < 0.01 vs. the corresponding control.

Because administration of *o*-Phen, Zn-Phen, and Rh-Phen at 5 µM was not cytotoxic to vascular endothelial cells [17], the effects of these compounds on proteoglycan expression in vascular endothelial cells was investigated using this concentration (Figure 1). After a 24-h incubation, *syndecan-4* mRNA levels were significantly increased by *o*-Phen (5.83-fold), Zn-Phen (3.17-fold), or Rh-Phen (1.71-fold) treatment.

**Figure 2:**
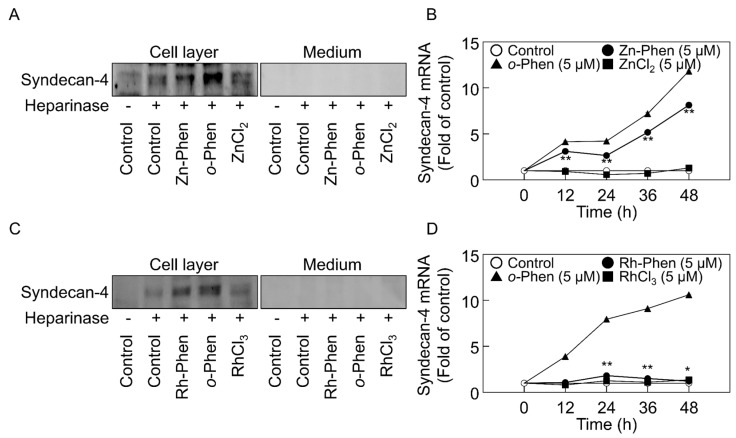
Effects of *o*-Phen, Zn-Phen, Rh-Phen, ZnCl_2_, and RhCl_3_ on syndecan-4 expression in vascular endothelial cells. Bovine aortic endothelial cells were treated with Zn-Phen, *o*-Phen, or ZnCl_2_ (**A**,**B**) and Rh-Phen, *o*-Phen, or RhCl_3_ (**C**,**D**) at 5 µM each at 37 °C for 24 h (**A**,**C**) or for 12, 24, 36, and 48 h (**B**,**D**). Syndecan-4 core protein and mRNA were analyzed by Western blot and real time RT-PCR, respectively. Values are means ± S.E.M. of four samples. * *p* < 0.05; ** *p* < 0.01 vs. control.

Figure 2 shows the expression of syndecan-4 core protein and mRNA in vascular endothelial cells after treatment with Zn-Phen, Rh-Phen, *o*-Phen, zinc chloride (ZnCl_2_), or rhodium (III) chloride (RhCl_3_). *o*-Phen (Figure 2A,C), Zn-Phen (Figure 2A), and Rh-Phen (Figure 2C) treatment increased syndecan-4 core-protein expression in the cell layer, whereas expression of the core protein was not detected in conditioned medium. However, both ZnCl_2_ (Figure 2A) and RhCl_3_ (Figure 2C) failed to increase endothelial syndecan-4 expression. Additionally, *syndecan-4* mRNA levels were significantly increased by *o*-Phen (Figure 2B,D), Zn-Phen (Figure 2B), and Rh-Phen (Figure 2C) treatment in a time-dependent manner, whereas this activity was not observed after treatment with ZnCl_2_ (Figure 2B) or RhCl_3_ (Figure 2D). Overall, we observed a significant response following *o*-Phen treatment, a moderate response to Zn-Phen treatment, and a weak response to Rh-Phen treatment (Figure 2B,D). Furthermore, the expression of endothelial syndecan-4 was induced by Zn-Phen or Rh-Phen treatment in a dose-dependent manner at ≥2 µM (Figure S1A–D). These results indicated that *o*-Phen induced endothelial syndecan-4 expression; however, the addition of zinc or rhodium to the molecular structure reduced this activity moderately and markedly, respectively.

### 2.2. The Hypoxia-Inducible Factor (HIF)-1α/β Pathway Specifically Mediates Upregulation of Syndecan-4 Expression by o-Phen and Zn-Phen

**Figure 3:**
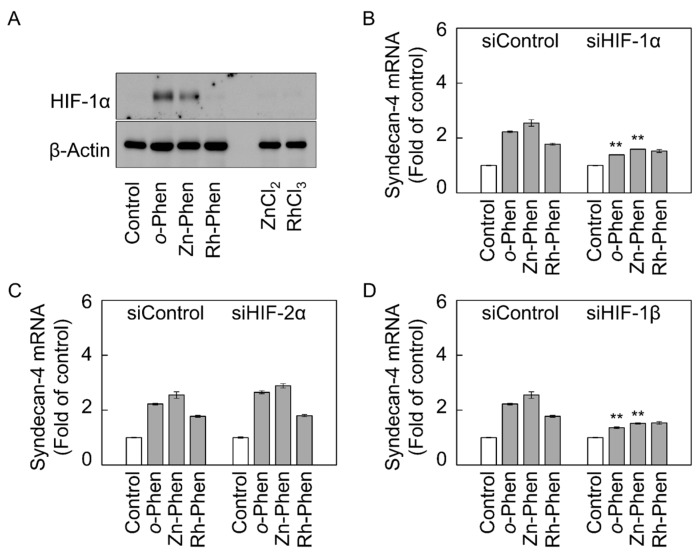
Involvement of hypoxia-inducible factor (HIF)-1α, HIF-2α, and HIF-1β in the induction of syndecan-4 expression by *o*-Phen, Zn-Phen, and Rh-Phen in vascular endothelial cells. (**A**) Bovine aortic endothelial cells were treated with *o*-Phen, Zn-Phen, Rh-Phen, ZnCl_2_, or RhCl_3_ at 5 µM each at 37 °C for 1 h, and HIF-1α expression was detected by Western blot analysis; (**B**) Effects of siRNA-mediated knockdown of *HIF-1α* on *syndecan-4* mRNA levels. Bovine aortic endothelial cells were transfected with siHIF-1α at 37 °C for 12 h and treated with *o*-Phen, Zn-Phen, or Rh-Phen at 5 µM each at 37 °C for 8 h; (**C**) Effects of siRNA-mediated knockdown of *HIF-2α* on *syndecan-4* mRNA levels. Bovine aortic endothelial cells were transfected with siHIF-2α at 37 °C for 12 h and treated with *o*-Phen, Zn-Phen, or Rh-Phen at 5 µM each at 37 °C for 8 h; (**D**) Effects of siRNA-mediated knockdown of *HIF-1β* on *syndecan-4* mRNA levels. Bovine aortic endothelial cells were transfected with siHIF-1β at 37 °C for 12 h and treated with *o*-Phen, Zn-Phen, or Rh-Phen at 5 µM each at 37 °C for 8 h. *Syndecan-4* mRNA was analyzed by real-time RT-PCR. Values are means ± S.E.M. of four samples. ** *p* < 0.01 vs. the corresponding siControl.

Because *o*-Phen induces hypoxia-inducible factor (HIF)-related protein expression [22], we investigated whether Zn-Phen and Rh-Phen also induce HIF-1α expression in vascular endothelial cells (Figure 3A). We observed that *o*-Phen or Zn-Phen treatment significantly increased the expression of HIF-1α protein; however, this was not observed following treatment with Rh-Phen, ZnCl_2_, or RhCl_3_.

To examine the involvement of HIF-1α in the induction of *syndecan-4* expression, vascular endothelial cells were transfected with *HIF-1α* small-interfering RNA (siRNA) and then treated with *o*-Phen, Zn-Phen, or Rh-Phen, followed by determination of *syndecan-4* mRNA levels. Our results showed that the *o*-Phen- or Zn-Phen-mediated induction of syndecan-4 expression was significantly suppressed by *HIF-1α* siRNA transfection (Figure 3B and Figure S2A). Although siRNA-mediated knockdown of *HIF-2α*, an isoform of the HIF-α subunit, did not influence *syndecan-4* mRNA levels (Figure 3C and Figure S2B), knockdown of *HIF-1β*, a HIF-α cofactor, significantly reduced *o*-Phen- or Zn-Phen-mediated induction of endothelial *syndecan-4* expression (Figure 3D and Figure S2C). Additionally, siRNA-mediated knockdown of aryl hydrocarbon receptor (*AhR*), which forms a complex with HIF-1β in the nucleus, failed to reduce *o*-Phen- or Zn-Phen-mediated induction of endothelial *syndecan-4* expression (Figure S3A,B), suggesting that AhR is not involved in this process. These findings suggested that induction of endothelial syndecan-4 expression by *o*-Phen or Zn-Phen treatment was mediated by the HIF-1α/β pathway without HIF-2α or AhR involvement.

### 2.3. o-Phen and Zn-Phen Inhibit Prolyl Hydroxylase Domain-Containing Protein 2 (PHD2) Activity

**Figure 4:**
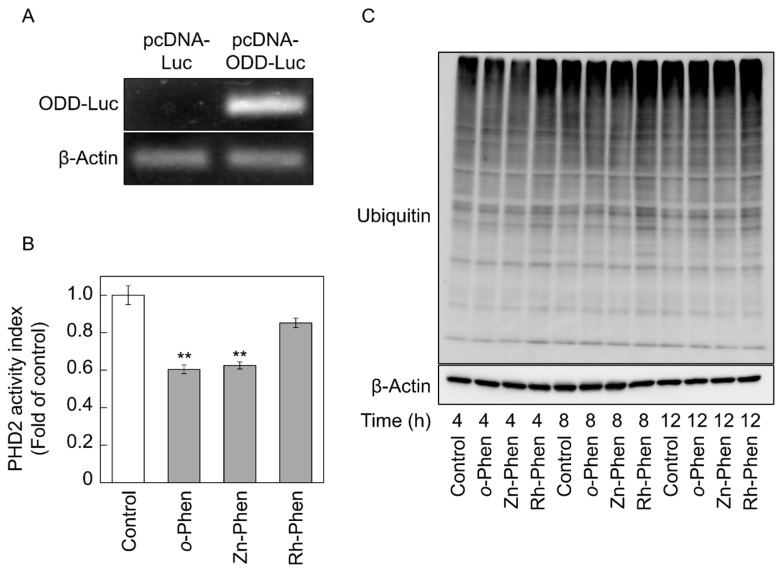
Effects of *o*-Phen, Zn-Phen, and Rh-Phen on prolyl hydroxylase domain-containing protein 2 (PHD2) activity in vascular endothelial cells. (**A**) Bovine aortic endothelial cells were transfected with pcDNA-Luc or pcDNA-ODD-Luc at 37 °C for 24 h. Amplified DNA products were separated by agarose gel electrophoresis and stained using ethidium bromide; (**B**) Bovine aortic endothelial cells were transfected with pcDNA-ODD-Luc at 37 °C for 12 h, treated with *o*-Phen, Zn-Phen, or Rh-Phen at 5 µM each at 37 °C for 24 h, and PHD2 activity was subsequently analyzed by luciferase assay. Values are means ± S.E.M. of five samples. ** *p* < 0.01 vs. control; (**C**) Bovine aortic endothelial cells were treated with *o*-Phen, Zn-Phen, or Rh-Phen at 5 µM each at 37 °C for 4, 8, and 12 h, and levels of ubiquitinated proteins were detected by Western blot analysis.

HIF-1α levels are regulated by its degradation via the ubiquitin-proteasome pathway following hydroxylation of HIF-1α Pro564 in the oxygen-dependent degradation domain (ODD) by prolyl hydroxylase domain-containing protein 2 (PHD2) [23]. We transfected a pcDNA-ODD-Luc vector into vascular endothelial cells (Figure 4A), followed by treatment with *o*-Phen, Zn-Phen, or Rh-Phen and measurement of PHD2 activity (Figure 4B). We observed significantly reduced PHD2 activity in the presence of *o*-Phen or Zn-Phen, but not Rh-Phen, and no changes in the amount of ubiquitinated proteins after treatment with *o*-Phen or Zn-Phen (Figure 4C), indicating that *o*-Phen- and Zn-Phen-mediated induction of HIF-1α expression was not due to stimulation of the ubiquitin-proteasome pathway.

## 3. Discussion

We previously demonstrated that the cytotoxicity of organic–inorganic hybrid molecules is not dependent upon molecule hydrophobicity or the cytotoxicity associated with intramolecular metals in their inorganic forms [18,19]. Specifically, the cytotoxicity of hybrid molecules having a certain molecular structure is caused by interactions between the molecular structure and the metal ion. It is likely that nontoxic biological activity is also dependent upon these interactions. In this study, we compared the effects of *o*-Phen, Zn-Phen, and Rh-Phen on the synthesis of proteoglycans in vascular endothelial cells and found that these hybrid molecules induced syndecan-4 expression in the order of *o*-Phen, Zn-Phen, and Rh-Phen. Syndecan-4 is a heparan sulfate proteoglycan involved in focal adhesion [24], wound healing [25], and as a modulating factor of protein kinase C-α signaling [26]. Although little is known about the regulation of syndecan-4 synthesis, studies confirmed its increased expression in vascular smooth muscle cells [27] and osteoblasts [28] and decreased expression in satellite cells [29] following exposure to FGF-2. Furthermore, in vascular endothelial cells, tumor necrosis factor-α [30,31], lipopolysaccharide, and interleukin-1β [25] induce syndecan-4 expression; however, the signaling pathways that mediate syndecan-4 expression remained unclear. Recently, it was reported that the HIF-1-PHD2 axis upregulates syndecan-4 expression in cultured nucleus pulposus cells [32], indicating that regulation of syndecan-4 expression can be mediated by the HIF-1α/β pathway. Additionally, there is a consensus hypoxia-response element (HRE) sequence (ACGTG) located −571 bp from the promoter region of the *syndecan-4* gene in bovine cells (at −1.6 kbp in human cells) according to National Center for Biotechnology Information (NCBI) data, suggesting that HIF-related proteins can regulate syndecan-4 expression. The results presented in this study using organic–inorganic hybrid molecules Zn-Phen, Rh-Phen, and their ligand *o*-Phen revealed that the HIF-1α/β pathway mediated the upregulation of syndecan-4 expression in vascular endothelial cells, suggesting that organic–inorganic hybrid molecules are an effective tool for analyzing biological systems.

Activation of the HIF-1α/β pathway by *o*-Phen and Zn-Phen is possibly due to lower levels of PHD2 activity. *o*-Phen has been used as a reagent to measure Fe(II) levels in environmental water due to its ability to chelate divalent metals [33]. Because PHD2 contains Fe(II) in its active site, we postulated that *o*-Phen extracts the Fe(II) from PHD2, thereby reducing PHD2-related activity. The induction of endothelial HIF-1α expression by Zn-Phen treatment was weaker than that observed after *o*-Phen treatment, and no induction of HIF-1α expression was observed following Rh-Phen treatment. There are coordinating bonds between a zinc ion and the ligand present in Zn-Phen molecules, and Rh-Phen molecules form covalent bonds between a rhodium ion and its ligand. Therefore, it is likely that the chelating activity of *o*-Phen against the Fe(II) from PHD2 is reduced by the presence of the zinc and rhodium ions to a moderate and strong degree, respectively. This might explain our observation of moderate and strong reductions in HIF-1α induction by *o*-Phen following treatment with Zn-Phen and Rh-Phen, respectively. By contrast, we also observed similar degrees of PHD2 inhibition by both *o*-Phen and Zn-Phen, indicating that the detailed mechanisms underlying the effects of *o*-Phen, Zn-Phen, and Rh-Phen on endothelial syndecan-4 expression remain to be elucidated. However, the differential effects of *o*-Phen, Zn-Phen, and Rh-Phen treatment on syndecan-4 expression may provide insight into important mechanisms involved in regulating syndecan-4 synthesis in vascular endothelial cells. Although the induction of syndecan-4 may result from the chelating capacity of *o*-Phen, the mechanism was postulated by comparison of the activity of *o*-Phen, Zn-Phen, and Rh-Phen on endothelial syndecan-4 induction. In that sense, our results supported the use of organic–inorganic hybrid molecules as useful tools for analyzing biological systems.

Either HIF-1α or HIF-2α forms a heterodimer with HIF-1β, and both HIF-1α/HIF-1β and HIF-2α/HIF-1β heterodimers recognize the same consensus HRE sequence in the promoter regions of hypoxia-inducible genes [34]. Here, we showed that HIF-1α, but not HIF-2α, was involved in *o*-Phen- and Zn-Phen-mediated induction of endothelial *syndecan-4* expression. Several studies reported the high selectivity of transcription factors bound to HREs in the promoter regions of hypoxia-inducible genes [35,36]. Additionally, a recent study showed that the intranuclear localization of HIF-1α differs from that of HIF-2α, and that the half-life of HIF-1α is much shorter than that of HIF-2α [37]. According to these reports, it is possible that epigenetic mechanisms or specific cofactors are involved in HIF-1α modulation of specific gene expression. Therefore, these reports support our findings that it is likely that *o*-Phen- and Zn-Phen-mediated induction of syndecan-4 expression involves HIF-1α, but not HIF-2α, in vascular endothelial cells.

Syndecan-4 was originally isolated from rat microvascular endothelial cells as an antithrombin III-binding molecule [7,38]. Previous reports showed that the concentration of heparan-sulfate chains at the vascular endothelial cell surface decreases under hypoxic conditions, whereas the relative synthesis of heparan-sulfate chains capable of binding antithrombin III increases under the same conditions [39]. Here, we observed that syndecan-4 expression in vascular endothelial cells was elevated by the HIF-1α/β pathway activated by lower levels of PHD2 activity, suggesting that hypoxic conditions caused increased expression of endothelial syndecan-4. We hypothesized that syndecan-4 expression is increased under these conditions, whereas the expression of other heparan-sulfate proteoglycans, such as perlecan, might be decreased in vascular endothelial cells under hypoxic conditions. In this study, we observed lower levels of perlecan mRNA in vascular endothelial cells following treatment with *o*-Phen, Zn-Phen, or Rh-Phen. Vascular endothelial cells under hypoxic conditions, such as those during angina, might promote syndecan-4 synthesis to prevent vascular occlusion by reinforcing anticoagulant activity, which is partly due to the activation of antithrombin III by heparan-sulfate chains associated with syndecan-4 in the vascular endothelium. 

## 4. Materials and Methods

### 4.1. Materials

Bovine aortic endothelial cells were purchased from Cell Applications (San Diego, CA, USA). Dulbecco’s modified Eagle medium (DMEM) and Ca^2+^- and Mg^2+^-free phosphate-buffered saline (PBS) were obtained from Nissui Pharmaceutical (Tokyo, Japan). Fetal bovine serum (FBS) was purchased from Biosera (Kansas City, MO, USA). The bicinchoninic acid protein assay kit, Lipofectamine RNAiMAX, and Lipofectamine LTX were purchased from Thermo Fisher Scientific (Waltham, MA, USA). *o*-Phen was purchased from Tokyo Chemical Industry (Tokyo, Japan), and Zn-Phen and Rh-Phen were synthesized as previously described [17]. ZnCl_2_ and RhCl_3_ were obtained from Wako Pure Chemical Industries (Osaka, Japan). TaKaRa Ex Taq and the restriction enzymes EcoRI, HindIII, and XbaI were purchased from Takara Bio (Shiga, Japan). NEBuilder HiFi DNA assembly cloning kit was purchased from New England Biolabs (Ipswich, MA, USA). Heparinase II (derived from *Flavobacterium heparinum*) and heparinase III (EC 4.2.2.8, derived from *F. heparinum*) were purchased from IBEX Technologies (Montreal, QC, Canada). Diethylaminoethyl-Sephacel (DEAE-Sephacel) was purchased from Sigma-Aldrich (St. Louis, MO, USA). Goat polyclonal antibody against syndecan-4 (N-19) was obtained from Santa Cruz Biotechnology (Santa Cruz, CA, USA). Mouse monoclonal antibody against HIF-1α (610958) was purchased from BD Biosciences (Franklin Lakes, NJ, USA). Mouse monoclonal antibody against β-actin was obtained from Wako Pure Chemical Industries. Rabbit polyclonal antibody against ubiquitin (UG9510) was purchased from Biomol GmbH (Hamburg, Germany). Horseradish peroxidase-conjugated anti-rabbit (#7074) and anti-mouse (#7076) IgG antibodies were obtained from Cell Signaling Technology (Beverly, MA, USA). Horseradish peroxidase-conjugated anti-goat IgG antibody (ab6885) was obtained from Abcam (Bristol, UK). Other reagents of the highest grade available were obtained from Nacalai Tesque (Kyoto, Japan).

### 4.2. Cell Culture and Treatments

Vascular endothelial cells were cultured in a humidified atmosphere of 5% CO_2_ at 37 °C in DMEM supplemented with 10% FBS until confluence or subconfluence. The medium was discarded, and the cells were used for experiments after washing twice with serum-free DMEM.

### 4.3. siRNA Transfection

Transfection of siRNAs was performed using Lipofectamine RNAiMAX according to manufacturer protocol. Briefly, annealed siRNA duplex and Lipofectamine RNAiMAX were dissolved in Opti-MEM in separate tubes and incubated for 5 min at room temperature, followed by mixing and incubation for 20 min at room temperature. Vascular endothelial cells were grown to near subconfluence in DMEM supplemented with 10% FBS and then incubated at 37 °C for 12 h in fresh DMEM supplemented with 10% FBS in the presence of the siRNA/Lipofectamine RNAiMAX mixture. The final concentrations of siRNA and Lipofectamine RNAiMAX were 18 nM and 0.09%, respectively. The medium was then changed to DMEM, and the cells were treated for 12, 24, 36, and 48 h with *o*-Phen, Zn-Phen, Rh-Phen, ZnCl_2_, or RhCl_3_. The sequences of sense and antisense siRNAs were as follows: bovine *HIF-1α* siRNA (siHIF-1α), 5′-GGGAUUAACUCAGUUUGAACUdTdT-3′ (sense) and 5′-UUCAAACUGAGUUAAUCCCAUdTdT-3′ (antisense); bovine *HIF-2α* siRNA (siHIF-2α), 5′-AUUUUUGAGGCUCAAGUUCUC-3′ (sense) and 5′-GAACUUGAGCCUCAAAAAUGG-3′ (antisense); bovine *HIF-1β* siRNA (siHIF-1β), 5′-GAACUCUUAGGAAAGAAUAUUdTdT-3′ (sense) and 5′-UAUUCUUUCCUAAGAGUUCCUdTdT-3′ (antisense); bovine *AhR* siRNA (siAhR), 5′-AUUAAGUCGGUCUCUAUGCCU-3′ (sense) and 5′-GCAUAGAGACCGACUUAAUAC-3′ (antisense). Negative control siRNA (siControl; Qiagen, Valencia, CA, USA) was used as a nonspecific sequence.

### 4.4. Real-Time RT-PCR

The monolayer of vascular endothelial cells was washed twice with Ca^2+^- and Mg^2+^-free PBS and lysed with QIAzol lysis reagent (Qiagen). A quarter volume of chloroform was mixed with the lysate, centrifuged, and the supernatant was harvested, followed by addition of 70% ethanol at a concentration of 52.5%. The suspension was centrifuged at 20,000× *g*, and the supernatant was discarded. The precipitate was suspended again in 70% ethanol and centrifuged at 20,000× *g*, followed by collection and drying of the precipitate containing total RNA. Complementary DNA was synthesized from the mRNA using a high-capacity cDNA reverse transcription kit (Applied Biosystems, Foster, CA, USA). Real-time PCR was performed using GeneAce SYBR qPCR mix α (Nippon Gene, Tokyo, Japan) with 1 ng/µL cDNA and 0.1 µM primers (Table 1) in a StepOnePlus real-time PCR system (Applied Biosystems). Levels of *perlecan*, *syndecan-1*, *syndecan-2*, *syndecan-3*, *syndecan-4*, *glypican-1*, *biglycan*, *decorin*, *HIF-1α*, *HIF-2α*, *HIF-1β*, *β2-microgloblin (B2M)*, and *β-actin* mRNAs were quantified using the relative standard curve method. When the experimental group included Rh-Phen treatment or siRNA transfection, the fold change in intensity value of the target gene was normalized to that of *B2M*. In the other cases, the fold change in intensity value of the target gene was normalized to that of β-actin.

### 4.5. Proteoglycan Core-Protein Extraction and Western Blot Analysis

Proteoglycans were extracted from the cell layer and the conditioned medium of vascular endothelial cells under dissociative conditions. Specifically, the conditioned medium was harvested, and solid urea was added at a concentration of 8 M. The cell layer was washed twice with Ca^2+^- and Mg^2+^-free PBS and lysed with 8 M urea cell-extract solution (pH 7.5) containing 120 mM 6-aminohexanoic acid, 12 mM benzamidine, 10 mM *N*-ethylmaleimide, 2 mM ethylenediaminetetraacetic acid (EDTA), 0.1 M phenylmethanesulfonyl fluoride, 0.1 M NaCl, 50 mM Tris base, and 2% Triton X-100. The extract was applied to a DEAE-Sephacel (0.3 mL of resin) column and washed four times with 0.25 M NaCl and 8 M urea buffer (pH 7.5) containing 2 mM EDTA, 0.5% Triton X-100, and 50 mM Tris base. Proteoglycans were eluted from the column with 0.9 mL 3 M urea buffer (pH 7.5) containing 2 mM EDTA, 0.5% Triton X-100, and 50 mM Tris base and precipitated with 3.5 volumes of 1.3% potassium acetate in 95% ethanol for 2 h at −20 °C; this precipitation step was repeated three times. Precipitated proteoglycans were dissolved with 0.02 IU/mL heparinase II/III in 100 mM Tris-HCl buffer (pH 7.0) containing 10 mM calcium acetate and 18 mM sodium acetate for 3 h at 37 °C to determine the core proteins of syndecan-4. The proteoglycans were lysed in sodium dodecyl sulfate (SDS) sample buffer (50 mM Tris-HCl buffer solution containing 2% SDS and 10% glycerol (pH 6.8)), followed by incubation at 95 °C for 3 min. Proteoglycans were separated by SDS-polyacrylamide gel electrophoresis on a 4% to 16% polyacrylamide gel and transferred onto a polyvinyl difluoride membrane (Immobilon-P; Millipore, Billerica, MA, USA) at 2 mA/cm^2^ for 1 h according to a previously described method [40]. Membranes were blocked with 5% skim milk in 20 mM Tris-HCl buffer solution (pH 7.5) containing 150 mM NaCl and 0.1% Tween 20 and incubated overnight with a primary antibody at 4 °C. The membranes were washed and incubated with horseradish peroxidase-conjugated secondary antibodies for 1 h at room temperature. Immunoreactive bands were visualized using enhanced chemiluminescence Western blot detection reagents (Chemi-Lumi One L; Nacalai, Kyoto, Japan) and scanned using a LAS 3000 Imager (Fujifilm, Tokyo, Japan). Representative blots are shown from two independent experiments.

### 4.6. Plasmid Construction

Firefly luciferase-reporter plasmid pGL4.12 (Promega, Fitchburg, WI, USA) was treated with restriction enzymes HindIII and XbaI, and the luciferase-containing fragments were inserted into the pcDNA3.1(+) vector (Invitrogen, Carlsbad, CA, USA) digested with the same two restriction enzymes to create a luciferase-containing pcDNA (pcDNA-Luc). Bovine HIF-1α ODD cDNA and stop-codon-insertion (STOP) cDNA were cloned from RT-PCR products from bovine vascular endothelial cells using TaKaRa Ex Taq and primers. ODD primers were designed using the NCBI database (Accession no. NM_174339_3) and the NEBuilder Assembly Tool (New England Biolabs). The sequences of the forward and reverse primers were as follows: ODD, 5′-CTGGCTAGCGTTTAAACTTAATGTTCAAGTTGGAATTGG-3′ and 5′-CCAACAGTACCGGATTGCCAAGGAGGTTCTTTAGGTACG-3′, respectively; STOP, 5′-GAGAATTCATAACACCCTCAAGATTGTCAGCAA-3′ and 5′-CACTCTAGAACAGTCTTCTGGGTGGCAGTGA-3′, respectively. The ODD PCR product was assembled using pcDNA-Luc digested with HindIII and using the NEBuilder HiFi DNA assembly cloning kit (New England Biolabs). The STOP PCR products digested with EcoRI and XbaI were inserted into the assembled plasmid digested with the same two restriction enzymes to create pcDNA-ODD-Luc. The DNA sequence of the pcDNA-ODD-Luc vector was analyzed by Eurofin Genomics (Tokyo, Japan). The mRNA expression from ODD-Luc was measured by real-time RT-PCR. The sequence of forward and reverse primers for ODD-Luc was 5′-CTGGCTAACTAGAGAACCCACTG-3′ and 5′-AGGGGCAAACAACAGATGG-3′, respectively. The amplicon was electrophoresed in a 1% agarose gel, stained with ethidium bromide, and visualized using a LAS 3000 Imager (Fujifilm).

### 4.7. Luciferase Assay

pcDNA-ODD-Luc and the *Renilla* luciferase-expression plasmid pRL-SV40 (Promega) were transfected into vascular endothelial cells using Lipofectamine LTX and PLUS reagents according to manufacturer instructions. Confluent cultures of vascular endothelial cells were prepared in 24-well culture plates. Plasmid vectors and PLUS reagent in Opti-MEM and Lipofectamine LTX in Opti-MEM were prepared in separate tubes and mixed, followed by incubation for 5 min at room temperature. Confluent vascular endothelial cells were prepared in fresh DMEM supplemented with 10% FBS, and the mixture was added to the medium. The final concentrations of pcDNA-ODD-Luc, pRL-SV40, Lipofectamine LTX, and PLUS reagent were 230 ng/mL, 0.46 ng/mL, 0.09%, and 0.04%, respectively. After a 12-h incubation, the medium was discarded, and the cells were incubated with 5 µM of *o*-Phen, Zn-Phen, or Rh-Phen. After 24 h, luciferase activity in the cells was measured using the dual-luciferase reporter assay and a GloMax 20/20n luminometer (Promega). The luminescent intensity value of firefly luciferase was normalized to that of *Renilla* luciferase, and the PHD2-activity index was calculated from the corresponding relative luciferase activity.

### 4.8. Statistical Analysis

Data were analyzed for statistical significance by analysis of variance and Student’s *t* test or Tukey’s method as appropriate. A *p* < 0.05 was considered statistically significant.

## 5. Conclusions

Our results showed that the application of organic–inorganic hybrid molecules may be an effective strategy for analyzing the regulatory mechanisms associated with vascular endothelial cells.

## Figures and Tables

**Table 1:** Bovine gene-specific primers for RT-PCR.

Gene	Forward Primer (5′–3′)	Reverse Primer (5’–3’)
*Perlecan*	ATGGCAGCGATGAAGCGGAC	TTGTGGACACGCAGCGGAAC
*Syndecan-1*	CAGTCAGGAGACAGCATCAG	CCGACAGACATTCCATACC
*Syndecan-2*	CCAGATGAAGAGGACACAAACG	CCAATAACTCCGCCAGCAA
*Syndecan-3*	CAAGCAGGCGAGCGTC	GGTGGCAGAGATGAAGTGG
*Syndecan-4*	TTGCCGTCTTCCTCGTGC	AGGCGTAGAACTCATTGGTGG
*Glypican-1*	GAAGGTCGGCAGGAAGAG	CCAGGAGCAGCAGAGGA
*Biglycan*	GCTGCCACTGCCATCTGAG	CGAGGACCAAGGCGTAG
*Decorin*	CTGCGGTTGACAATGGC	CTCACTCCTGAATAAGAAGCC
*HIF-1α*	GCTTGCTCATCAGTTGCCAC	GCATCCAGAAGTTTCCTCACAC
*HIF-2α*	CAGTGGCAAGGTGGCTGTGTC	GGTCCCGAAATCCAGAGAAATGA
*HIF-1β*	TAAGGAGCGGTTTGCCAGGTC	TTCTGTTATGTAGGCTGTCATCTTGTTC
*AhR*	GTGTCAGTTATCTCAGAGCCAAG	AAAGCCATTTAGTGCCTGTAGTA
*B2M*	CCATCCAGCGTCCTCCAAAGA	TTCAATCTGGGGTGGATGGAA
*β-Actin*	CCTCCCTGGAGAAGAGCTACGA	GGAATTGAAGGTAGTTTCGTGAATG

## Supplementary Materials

Supplementary materials can be found at www.mdpi.com/1422-0067/18/2/352/s1.

## Abbreviations

AhR	Aryl hydrocarbon receptor
B2M	β2-Microgloblin
DMEM	Dulbecco’s modified Eagle medium
PBS	Phosphate-buffered saline
FBS	Fetal bovine serum
HIF	Hypoxia-inducible factor
FGF-2	Fibroblast growth factor-2
HRE	Hypoxia-response element
ODD	Oxygen-dependent degradation domain
*o*-Phen	1,10-Phenanthroline
PHD2	Prolyl hydroxylase domain-containing protein 2
Rh-Phen	Potassium tetrachloro(1,10-phenanthroline)rhodate(III)
siRNA	Small interfering RNA
Zn-Phen	Dichloro(1,10-phenanthroline)zinc

## References

[1] Jaffe E.A., Hoyer L.W., Nachman R.L. (1974). Synthesis of von Willebrand factor by cultured human endothelial cells. Proc. Natl. Acad. Sci. USA.

[2] Maynard J.R., Dreyer B.E., Stemerman M.B., Pitlick F.A. (1977). Tissue-factor coagulant activity of cultured human endothelial and smooth muscle cells and fibroblasts. Blood.

[3] Revtyak G.E., Johnson A.R., Campbell W.B. (1987). Prostaglandin synthesis in bovine coronary endothelial cells: Comparison with other commonly studied endothelial cells. Thromb. Res..

[4] Levin E.G., Loskutoff D.J. (1982). Cultured bovine endothelial cells produce both urokinase and tissue-type plasminogen activators. J. Cell Biol..

[5] Ruoslahti E. (1988). Structure and biology of proteoglycans. Annu. Rev. Cell Biol..

[6] Saku T., Furthmayr H. (1989). Characterization of the major heparan sulfate proteoglycan secreted by bovine aortic endothelial cells in culture. Homology to the large molecular weight molecule of basement membranes. J. Biol. Chem..

[7] Kojima T., Shworak N.W., Rosenberg R.D. (1992). Molecular cloning and expression of two distinct cDNA-encoding heparan sulfate proteoglycan core proteins from a rat endothelial cell line. J. Biol. Chem..

[8] Mertens G., Cassiman J.J., Van den Berghe H., Vermylen J., David G. (1992). Cell surface heparan sulfate proteoglycans from human vascular endothelial cells. Core protein characterization and antithrombin III binding properties. J. Biol. Chem..

[9] Schönherr E., O’Connell B.C., Schittny J., Robenek H., Fastermann D., Fisher L.W., Plenz G., Vischer P., Young M.F., Kresse H. (1999). Paracrine or virus-mediated induction of decorin expression by endothelial cells contributes to tube formation and prevention of apoptosis in collagen lattices. Eur. J. Cell Biol..

[10] Yamamoto C., Deng X., Fujiwara Y., Kaji T. (2005). Proteoglycans predominantly synthesized by human brain microvascular endothelial cells in culture are perlecan and biglycan. J. Health Sci..

[11] Tollefsen D.M., Pestka C.A., Monafo W.J. (1983). Activation of heparin cofactor II by dermatan sulfate. J. Biol. Chem..

[12] Kaji T., Yamamoto C., Oh-i M., Fujiwara Y., Yamazaki Y., Morita T., Plaas A.H., Wight T.N. (2006). The vascular endothelial growth factor VEGF165 induces perlecan synthesis via VEGF receptor-2 in cultured human brain microvascular endothelial cells. Biochim. Biophys. Acta.

[13] Kinsella M.G., Tsoi C.K., Jarvelainen H.T., Wight T.N. (1997). Selective expression and processing of biglycan during migration of bovine aortic endothelial cells. The role of endogenous basic fibroblast growth factor. J. Biol. Chem..

[14] Kaji T., Yamamoto C., Oh-i M., Nishida T., Takigawa M. (2004). Differential regulation of biglycan and decorin synthesis by connective tissue growth factor in cultured vascular endothelial cells. Biochem. Biophys. Res. Commun..

[15] Kaji T., Yamada A., Miyajima S., Yamamoto C., Fujiwara Y., Wight T.N., Kinsella M.G. (2000). Cell density-dependent regulation of proteoglycan synthesis by transforming growth factor-β_1_ in cultured bovine aortic endothelial cells. J. Biol. Chem..

[16] Hara T., Yoshida E., Shinkai Y., Yamamoto C., Fujiwara Y., Kumagai Y., Kaji T. (2016). Biglycan intensifies ALK5-Smad2/3 signaling by TGF-β1 and downregulates syndecan-4 in cultured vascular endothelial cells. J. Cell. Biochem..

[17] Hara T., Matsuzaki H., Nakamura T., Yoshida E., Ohkubo T., Maruyama H., Yamamoto C., Saito S., Kaji T. (2016). Cytotoxicity of zinc, copper and rhodium complexes with 1,10-phenanthroline or 2,9-dimethyl-1,10-phenanthroline in cultured vascular endothelial cells. Fundam. Toxicol. Sci..

[18] Kohri K., Yoshida E., Yasuike S., Fujie T., Yamamoto C., Kaji T. (2015). The cytotoxicity of organobismuth compounds with certain molecular structures can be diminished by replacing the bismuth atom with an antimony atom in the molecules. J. Toxicol. Sci..

[19] Murakami M., Fujie T., Matsumura M., Yoshida E., Yamamoto C., Fujiwara Y., Yasuike S., Kaji T. (2015). Comparative cytotoxicity of triphenylstibane and fluorine-substituted triarylpnictogens in cultured vascular endothelial cells. Fundam. Toxicol. Sci..

[20] Fujie T., Murakami M., Yoshida E., Yasuike S., Kimura T., Fujiwara Y., Yamamoto C., Kaji T. (2016). Transcriptional induction of metallothionein by tris(pentafluorophenyl)stibane in cultured bovine aortic endothelial cells. Int. J. Mol. Sci..

[21] Fujie T., Segawa Y., Yoshida E., Kimura T., Fujiwara Y., Yamamoto C., Satoh M., Naka H., Kaji T. (2016). Induction of metallothionein isoforms by copper diethyldithiocarbamate in cultured vascular endothelial cells. J. Toxicol. Sci..

[22] Cho E.A., Song H.K., Lee S.H., Chung B.H., Lim H.M., Lee M.K. (2013). Differential in vitro and cellular effects of iron chelators for hypoxia inducible factor hydroxylases. J. Cell. Biochem..

[23] Ivan M., Kondo K., Yang H., Kim W., Valiando J., Ohh M., Salic A., Asara J.M., Lane W.S., Kaelin W.G. (2001). HIFα targeted for VHL-mediated destruction by proline hydroxylation: Implications for O_2_ sensing. Science.

[24] Whiteford J.R., Behrends V., Kirby H., Kusche-Gullberg M., Muramatsu T., Couchman J.R. (2007). Syndecans promote integrin-mediated adhesion of mesenchymal cells in two distinct pathways. Exp. Cell Res..

[25] Vuong T.T., Reine T.M., Sudworth A., Jenssen T.G., Kolset S.O. (2015). Syndecan-4 is a major syndecan in primary human endothelial cells in vitro, modulated by inflammatory stimuli and involved in wound healing. J. Histochem. Cytochem..

[26] Kwon S., Son H., Choi Y., Lee J.H., Choi S., Lim Y., Han I.O., Oh E.S. (2009). Syndecan-4 promotes the retention of phosphatidylinositol 4,5-bisphosphate in the plasma membrane. FEBS Lett..

[27] Cizmeci-Smith G., Langan E., Youkey J., Showalter L.J., Carey D.J. (1997). Syndecan-4 is a primary-response gene induced by basic fibroblast growth factor and arterial injury in vascular smooth muscle cells. Arterioscler. Thromb. Vasc. Biol..

[28] Song S.J., Cool S.M., Nurcombe V. (2007). Regulated expression of syndecan-4 in rat calvaria osteoblasts induced by fibroblast growth factor-2. J. Cell. Biochem..

[29] Velleman S.G., Li X., Coy C.S., McFarland D.C. (2008). The effect of fibroblast growth factor 2 on the in vitro expression of syndecan-4 and glypican-1 in turkey satellite cells. Poult. Sci..

[30] Okuyama E., Suzuki A., Murata M., Ando Y., Kato I., Takagi Y., Takagi A., Murate T., Saito H., Kojima T. (2013). Molecular mechanisms of syndecan-4 upregulation by TNF-α in the endothelium-like EAhy926 cells. J. Biochem..

[31] Zhang Y., Pasparakis M., Kollias G., Simons M. (1999). Myocyte-dependent regulation of endothelial cell syndecan-4 expression. Role of TNF-α. J. Biol. Chem..

[32] Fujita N., Hirose Y., Tran C.M., Chiba K., Miyamoto T., Toyama Y., Shapiro I.M., Risbud M.V. (2014). HIF-1-PHD2 axis controls expression of syndecan 4 in nucleus pulposus cells. FASEB J..

[33] Hoshi S., Yamada M., Inoue S., Matsubara M. (1989). Simple and rapid spectrophotometric determination of iron after preconcentration as its 1,10-phenanthroline complex on the natural polymer “chitin”. Talanta.

[34] Wenger R.H. (2002). Cellular adaptation to hypoxia: O_2_-sensing protein hydroxylases, hypoxia-inducible transcription factors, and O_2_-regulated gene expression. FASEB J..

[35] Mole D.R., Blancher C., Copley R.R., Pollard P.J., Gleadle J.M., Ragoussis J., Ratcliffe P.J. (2009). Genome-wide association of hypoxia-inducible factor (HIF)-1α and HIF-2α DNA binding with expression profiling of hypoxia-inducible transcripts. J. Biol. Chem..

[36] Schodel J., Oikonomopoulos S., Ragoussis J., Pugh C.W., Ratcliffe P.J., Mole D.R. (2011). High-resolution genome-wide mapping of HIF-binding sites by ChIP-seq. Blood.

[37] Taylor S.E., Bagnall J., Mason D., Levy R., Fernig D.G., See V. (2016). Differential sub-nuclear distribution of hypoxia-inducible factors (HIF)-1 and -2 α impacts on their stability and mobility. Open Biol..

[38] Shworak N.W., Shirakawa M., Colliec-Jouault S., Liu J., Mulligan R.C., Birinyi L.K., Rosenberg R.D. (1994). Pathway-specific regulation of the synthesis of anticoagulantly active heparan sulfate. J. Biol. Chem..

[39] Karlinsky J.B., Rounds S., Farber H.W. (1992). Effects of hypoxia on heparan sulfate in bovine aortic and pulmonary artery endothelial cells. Circ. Res..

[40] Kyhse-Andersen J. (1984). Electroblotting of multiple gels: A simple apparatus without buffer tank for rapid transfer of proteins from polycrylamide to nitrocellulose. J. Biochem. Biophys. Methods.

