# Supplementary material for: Induction of Syndecan-4 by Organic–Inorganic Hybrid Molecules with a 1,10-Phenanthroline Structure in Cultured Vascular Endothelial Cells
Source: Int J Mol Sci. 2017 Feb 8;18(2):352. doi: 10.3390/ijms18020352 (PMC5343887; doi:10.3390/ijms18020352)
Supplement: Supplementary file 1 [file ijms-18-00352-s001.pdf]

Supplementary Materials: Induction of Syndecan-4 by Organic-Inorganic Hybrid Molecules with a 1,10-Phenanthroline Structure in Cultured Vascular Endothelial Cells

Takato Hara, Takayuki Kojima, Hiroka Matsuzaki, Takehiro Nakamura, Eiko Yoshida, Yasuyuki Fujiwara, Chika Yamamoto, Shinichi Saito and Toshiyuki Kaji

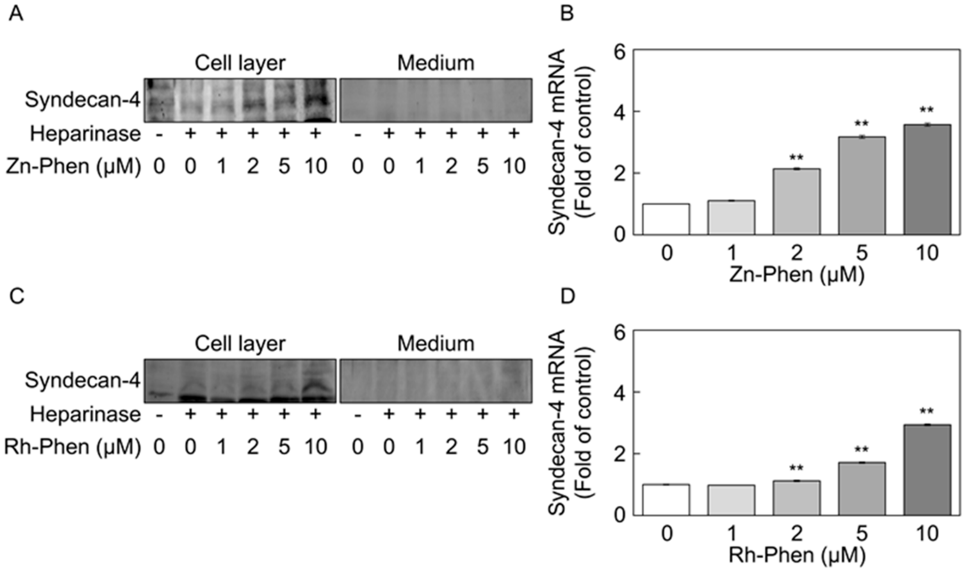

Figure S1. Effects of Zn-Phen and Rh-Phen on syndecan-4 expression of in vascular endothelial cells. Bovine aortic endothelial cells were treated with Zn-Phen (**A**,**B**) or Rh-Phen (**C**,**D**) at 1, 2, 5, or 10 μM each at 37 °C for 24 h. Syndecan-4 core protein and mRNA were analyzed by Western blot and real time RT-PCR respectively Values are means ± S.E.M. of four samples. ** $p < 0.01$ vs. the corresponding control.

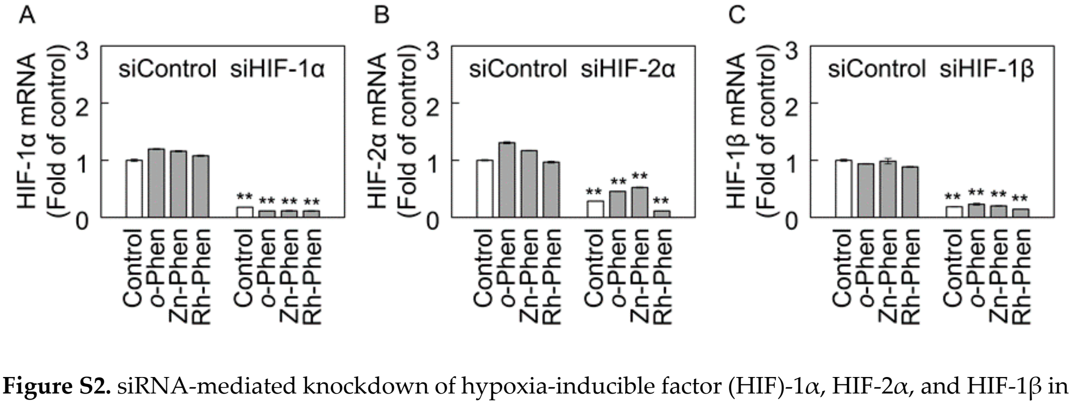

Figure S2. siRNA-mediated knockdown of hypoxia-inducible factor (HIF)-1α, HIF-2α, and HIF-1β in vascular endothelial cells. Bovine aortic endothelial cells were transfected with (**A**) siHIF-1α; (**B**) siHIF-2α, or (**C**) siHIF-1β, and the mRNA levels of the corresponding HIF proteins were determined by real time RT-PCR. Values are means ± S.E.M. of four samples. ** $p < 0.01$ vs. the corresponding siControl.

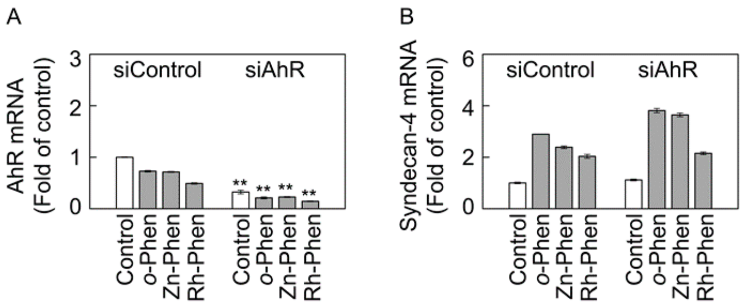

Figure S3. Involvement of aryl hydrocarbon receptor (AhR) in the induction of syndecan-4 expression by *o*-Phen, Zn-Phen, and Rh-Phen in vascular endothelial cells. Bovine aortic endothelial cells were transfected with siAhR at 37 °C for 12 h and treated with *o*-Phen, Zn-Phen, or Rh-Phen at 5 μM each at 37 °C for 8 h. (**A**) AhR mRNA levels; (**B**) syndecan-4 mRNA levels were analzed by real time RT-PCR. Values are means ± S.E.M. of four samples. ** $p < 0.01$ vs. the corresponding siControl.
